# Supplementary figures and images for: Cross-sectional study of asymptomatic Neisseria gonorrhoeae and Chlamydia trachomatis infections in sexually transmitted disease related clinics in Shenzhen, China
Source: PLoS One. 2020 Jun 9;15(6):e0234261. doi: 10.1371/journal.pone.0234261 (PMC7282648; doi:10.1371/journal.pone.0234261)

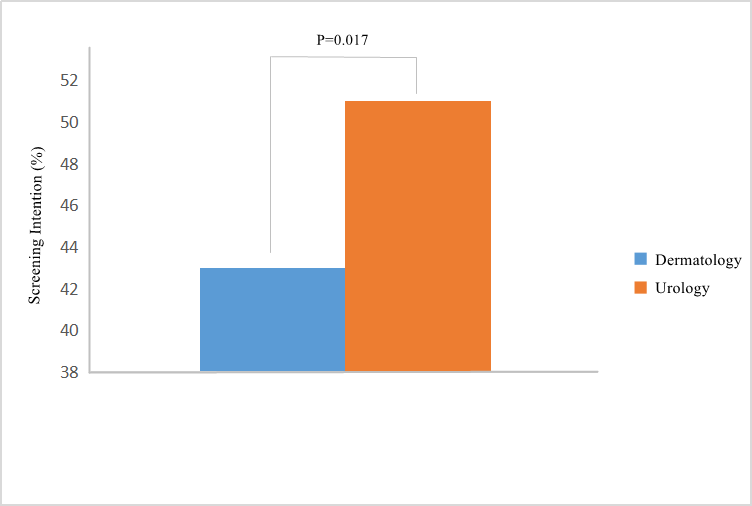

Supplement: S1 Fig — (TIFF) [file pone.0234261.s001.tiff]
