# Supplementary material for: Cross-sectional study of asymptomatic Neisseria gonorrhoeae and Chlamydia trachomatis infections in sexually transmitted disease related clinics in Shenzhen, China
Source: PLoS One. 2020 Jun 9;15(6):e0234261. doi: 10.1371/journal.pone.0234261 (PMC7282648; doi:10.1371/journal.pone.0234261)
Supplement: S1 Questionnaire — (DOC) [file pone.0234261.s002.doc]

# 生殖道沙眼衣原体检测知情同意书

您好！

近年来，我市生殖道沙眼衣原体感染报告发病率迅速上升，给广大人民群众带来严重的疾病负担并危害生殖健康。本筛查作为深圳市淋病和生殖道沙眼衣原体感染综合防治项目内容之一，系由深圳市慢性病防治中心组织，在全市范围内实施的一项公共卫生服务项目。开展沙眼衣原体检测，能及时发现您的感染现状，并及时提供规范治疗。有关本次调查情况，请您仔细阅读以下内容。感谢您的配合！

1、本次调查将为您提供免费的生殖道沙眼衣原体检测服务；

2、本次调查将帮助您及时发现可能的生殖道沙眼衣原体感染，并提供规范治疗和干预。

3、在采集标本的过程中，您可能会稍微感到不适，但对您的正常生活不会产生任何影响；

4、本次检测过程中您提供的个人信息将严格保密；

5、本次检测完全自愿。

感谢您的配合与支持！如有任何疑问，请拨打电话咨询：

**深圳市慢性病防治中心性病防治科：25632714**

□我已充分了解本次调查相关内容，**同意**参加本次调查。

□我已充分了解本次调查相关内容，**不同意**参加本次调查。

**调查者签名 调查日期: 年 月 日**

**附件8**

**贴编码处**

**深圳市诊疗机构门诊就诊者信息采集表**

您好！

近年来，我市淋病和生殖道沙眼衣原体感染报告发病率迅速上升，给广大人民群众带来严重疾病负担并危害生殖健康。深圳市淋病和生殖道沙眼衣原体感染综合防治项目系由深圳市卫生和计划生育委员会组织，深圳市慢性病防治中心在全市范围内实施的一项公共卫生服务项目。开展淋病和沙眼衣原体检测，能及时发现您的感染现状，并及时提供规范治疗。

感谢您的配合与支持！如有任何疑问，请拨打电话咨询：

**深圳市慢性病防治中心性病防治科：25632714**

**纳入标准：**①年龄在18-49岁；②有过性行为；③最近2周内没有使用过抗生素者。

皮肤性病科就诊者还需满足以下条件之一：①具有性病可疑症状；②感染其它性病；③性伴患有性病；④具有高危性行为，如有多性伴性行为或与感染状态不明的新性伴发生了不安全性行为；⑤男性同性恋门诊就诊者。

01 医院名称（hospital）：___________

02 就诊科室（office）：①皮肤（性病）科 ②妇科 ③泌尿外科 ④肛肠科 ⑤其他

03 性别（sex）：①男 ②女

04 年龄（age）：______岁

05 婚姻状况（marriage）：①未婚 ②已婚 ③离异 ④丧偶

06 夫妻分居情况（separation）：①是 ②否

07 生育情况（dilivery）：①无子女 ②有子女 ③其他______

08 户口所在地（hukou）：①深圳 ②非深圳

09 在深圳居住时间（residetime）：①＜3月 ②3~6月 ③7~12月 ④1 ~2年 ⑤≥2年

10 职业（occupation）（在相应数字处打√））：

①工人 ②娱乐服务 ③商业服务 ④公务员 ⑤企事业单位职员

⑥家庭主妇 ⑦餐饮服务 ⑧失业/待业 ⑨其他________

11 文化程度（education）：①小学及以下 ②初中 ③高中/中专 ④大专 ⑤本科 ⑥硕士及以上

12 是否使用医疗保险（insurance）：

①是（insurother）（①社会医疗保险 ②劳务工医疗保险 ③其他医疗保险） ②否

13 疾病认识（know）：您对生殖道沙眼衣原体感染有什么样的了解？**（多选题）**

①不了解 ②是一种传染病 ③是一种生殖道感染疾病

④是一种通过性接触传播的疾病 ⑤其他：

14 疾病看法（attitude）：您认为生殖道沙眼衣原体感染对人体的危害如何？**（多选题）**

①没有任何危害 ②对性生活有影响 ③对生育能力有影响 ④不了解

15 检测经历（testhistroy）：您既往有过生殖道沙眼衣原体感染检测经历吗？①没有 ②有

16 感染经历（infecthistory）：您曾经被诊断为生殖道沙眼衣原体感染及接受过治疗吗？

①没有 ②诊断并治疗过 ③诊断但未治疗 ④不知道/记不清

17 筛查看法（screena）：如果需要您每年做一次衣原体检测，您愿意接受检测吗？

①不愿意 ②只有我发现自己有不舒服才愿意（需要）检测

③如果是免费我就愿意 ④无论是否免费我都愿意 ⑤其他：

18 筛查看法（screenb）：如果发现您有生殖道沙眼衣原体感染，是否愿意告知您的性伴？

①不愿意，担心： ②愿意 ➂其他：

19 患者目前有下列哪些症状和体征（可填多项，在“□”和相应数字处打“√”）：

| **女性（FP）** |  | **男性（MP）** |
| --- | --- | --- |
| 阴道分泌物增多或有异味 | 1 | 尿道有浆液性分泌物 |
| 尿急、尿痛、尿道口红肿、溢脓 | 2 | 尿道有脓性分泌物 |
| 白带异常 | 3 | 尿频、尿急、尿痛，尿道瘙痒 |
| 宫颈充血、黏液脓性分泌物 | 4 | 阴囊肿胀、疼痛 |
| 下腹痛 | 5 | 附睾疼痛，肿大，触痛 |
| □生殖器 □肛周长有水疱 | 6 | □生殖器 □肛周有水疱、脓疱 |
| □生殖器 □肛周有破溃/溃疡 | 7 | □生殖器 □肛周有破溃/溃疡 |
| □生殖器 □肛周有疣状赘生物 | 8 | □生殖器 □肛周有疣状赘生物 |
| 手、足心部位长有红斑 | 9 | 手、足心部位长有红斑 |
| 腹股沟淋巴结肿大 | 10 | 腹股沟淋巴结肿大 |
| 其他______________(请注明) | 11 | 其他______________(请注明) |
| **无症状/体征** | 12 | **无症状/体征** |

20 月收入（salary）：______元；配偶的月收入（salaryb）：______元

21 性取向（orientation）：①异性 ②同性 ③双性 ④其他______

22 最近3月内，您是否和**配偶或男/女朋友以外**的人发生过性行为（sexother）：①是 ②否

**采集样本时请注意核对样本采集管、拭子管编号与本信息表编号一致。**

就诊日期： 年 月 日 医生签名：____________ 质控员：____________

**深圳市诊疗机构门诊患者信息采集表**

数据库编码查询

1、xqbm（辖区编码）：①罗湖区，②南山区，③宝安区，④龙岗区，⑤盐田区，⑥龙华区。

2、office（科室）：①皮肤性病科，②妇科，③泌尿外科，④肛肠科，⑤其他科。

3、sex（性别）：①男，②女。

4、agegp（年龄分组）：①≤24岁，②＞24岁。

5、marriage（婚姻状况）：①未婚，②已婚，③离异，④丧偶。

6、separation（夫妻分居）：①是，②否。

7、orientation（性取向）：①异性，②同性，③双性，④离异。

8、dilivery（生育情况）：①无子女，②有子女，③其他。

9、hukou（户口所在地）：①深圳，②非深圳。

10、residetime（在深圳居住时间）：①＜3月，②3-6月，③7-12月，④1-2年，⑤≥2年。

11、occupation（职业）：①工人，②娱乐服务，③商业服务，④公务员，⑤企事业单位，⑥家庭主妇，⑦餐饮服务，⑧失业/待业，⑨其他。

12、salarygp（月收入）：①＜3千元，②3-5千元，③5-10千元，④≥1万元。（salaryb同样）

13、education（文化程度）：①小学及以下，②初中，③高中/中专，④大专，⑤本科，⑥硕士及以上。

14、insurance（医疗保险）：①是，②否。

15、know（疾病认知）：①不了解，②是一种传染病，③是一种生殖道感染疾病，④是一种通过性接触传播的疾病，⑤其他。

16、attitude（疾病看法）：①没有任何危害，②对性生活有影响，③对生育能力有影响，④不了解。

17、testhistory（检测经历）：①没有，②有。

18、infecthistory（感染经历）：①没有，②诊断并治疗过，③诊断但未治疗，④不知道/记不清。

19、screena（是否愿意CT检测）：①不愿意，②只有我发现自己阳性有不舒服才愿意检测，③如果免费就愿意，④无论是否免费都愿意，⑤其他。

20、screenb（CT阳性是否告知性伴）：①不愿意，②愿意，③其他。

21、FP（女性患者有无症状和体征）、MP（男性患者有无症状和体征）：①是，②否。

22、sexother（最近3个月内是否和配偶或男/女朋友以外的人发生过性行为）：①是，②否。

23、NG、NGtest（淋球菌检测结果）：0阴性，①阳性。

24、CT、CTtest（沙眼衣原体感染检测结果）：0阴性，①阳性。
