# Supplementary material for: Cross-sectional study of asymptomatic Neisseria gonorrhoeae and Chlamydia trachomatis infections in sexually transmitted disease related clinics in Shenzhen, China
Source: PLoS One. 2020 Jun 9;15(6):e0234261. doi: 10.1371/journal.pone.0234261 (PMC7282648; doi:10.1371/journal.pone.0234261)
Supplement: Attachment — Submitted filename: Chang et al reviewers comments PLOSONE.docx [file pone.0234261.s004.docx]

Thank you for the opportunity to review your interesting and important research

Major comments

1. The aim of the study was to determine prevalence of asymptomatic *Chlamydia trachomatis* and *Neisseria gonorrhoeae* infections and the risk factors associated with these infections. However, as the authors have already conceded that due to low a prevalence of *N. gonorrhoeae* in their study, the number of *C. trachomatis* and *N. gonorrhoeae* cases were combined therefore the risks factors associated with asymptomatic infection of each pathogen could not be independently determined. Could you discuss in your limitations the implications of this? I think the title of the study in that regard does not address the aims as the risk factors associated with asymptomatic *C. trachomatis* and *N. gonorrhoeae* were not independently determined in this study.

2. The authors determined one the risk factors for asymptomatic *C. trachomatis* / *N. gonorrhoeae* was recruitment from urology department (men) and dermatology (men). Did you include results from the other 46 departments in your analysis? In your results in Table 3 and 5 data from only three departments is shown.

3. Results in table 2 and table 3 should also include the actual number of positive *C. trachomatis* and *N. gonorrhoeae* cases not just as “positive cases”

Minor comments

Line 42 and 80: “Chlamydia trachomatis” and “Neisseria gonorrhoeae” should always be in italics

Line 82: can you cite a more recent reference with up-to-date epidemiological information

Line 86: please add a reference to support mentioned statements

Line 150: “magnetism” should be “magnetic”

Line 264: “but these two diseases (especially CT)” please rephrase as CT and NG are bacterial names not diseases

Results: were there any participants with dual *C. trachomatis* or *N. gonorrhoeae* infections?
